# Second Case of Tumors Associated With Heterozygous NTHL1 Variant

**DOI:** 10.7759/cureus.26734

**Published:** 2022-07-11

**Authors:** Danyon J Anderson, Trenton Reinicke, Andrew W Boyle, Mokshal H Porwal, Allan H Friedman

**Affiliations:** 1 Medicine, Medical College of Wisconsin, Wauwatosa, USA; 2 Gastroenterology, Massachusetts General Hospital, Boston, USA; 3 Research, California Institute of Technology, Pasadena, USA; 4 Neurological Surgery, Duke University, Durham, USA

**Keywords:** invasive ductal cell carcinoma, papillary carcinoma of thyroid, meningioma, pilocytic astrocytoma, gastrointestinal stromal tumor (gist), heterozygous, nthl1 tumor syndrome, nthl1

## Abstract

Homozygous mutations to *NTHL1 *are known to increase cancer risk, particularly in the colon and breast. *NTHL1 *tumor syndrome (NTS) is an autosomal recessive genetic condition. Little is known about the cancer risk in patients who have heterozygous *NTHL1 *mutations. We previously published a case of benign tumors associated with a heterozygous *NTHL1 *mutation. In this second case, we present a patient with a heterozygous *NTHL1 *mutation who developed a gastrointestinal stromal tumor, pilocytic astrocytoma, tall cell papillary thyroid cancer, invasive ductal papilloma, spinal nerve sheath tumors, and spinal hemangiomas. Here, we show that heterozygous *NTHL1 *mutations may increase cancer risk and may even manifest similarly to NTS.

## Introduction

*NTHL1* is a gene that encodes for a DNA glycosylase involved in DNA base excision repair [1]. *NTHL1* tumor syndrome (NTS) is autosomal polyposis that is inherited recessively [1-5]. It is associated with colon and breast cancer [1-5]. *NTHL1* codes for a protein that repairs DNA through base excision [6,7]. Although patients who are homozygous for variants of *NTHL1* have developed more than a dozen tumor types affecting more than a half dozen organs, no large studies have analyzed the risk of being heterozygote for *NTHL1* variants on tumorigenesis [2]. Among the limited literature on heterozygous *NTHL1* mutations, one case report found spinal schwannoma, arm schwannoma, and hepatic hemangioma in a patient with a heterozygous *NTHL1* mutation [8]. In this report, we present a second patient with a heterozygous variant of *NTHL1* with a history of recurrent tumorigenesis.

## Case presentation

A 29-year-old woman without a significant past medical history presented to an emergency room with abdominal pain. A CT scan showed perforated appendicitis. The patient underwent emergency surgery during which a gastrointestinal stromal tumor (GIST) and appendiceal carcinoma were removed.

Three years later, the patient suffered from intense headaches that went untreated for a year at which point she had focal seizures affecting right motor function while driving. Brain MRI showed a T1 contrast-enhancing mass next to a non-enhancing cyst in her cerebellum causing hydrocephalus. Gross total resection of the mass was undertaken, and pathology confirmed the mass to be a pilocytic astrocytoma. Her hydrocephalus was treated with a ventriculostomy. Brain imaging incidentally identified neck nodules.

Five years after the initial presentation, a thyroid biopsy was performed due to radiologic findings and past tumor history. Biopsy demonstrated tall cell papillary thyroid cancer. The patient was treated by neck dissection and adjuvant radioactive iodine. Six years after the initial presentation, she sought medical care for heavy menstrual bleeding and was diagnosed with uterine leiomyoma, which was removed by hysteroscopy.

**Figure 1:**
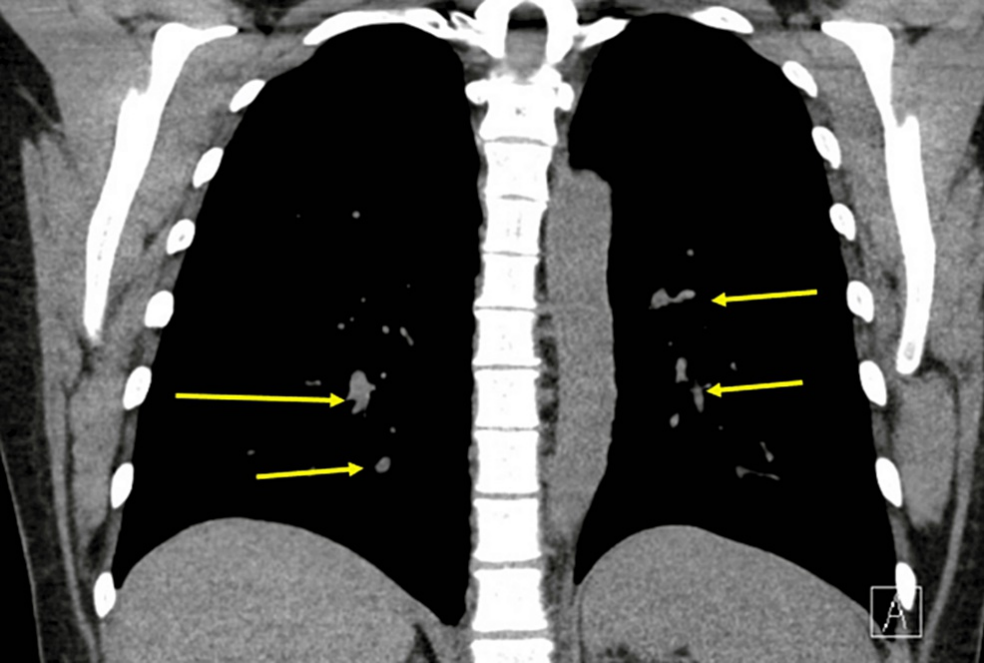
CT chest showing possibly metastatic pulmonary nodules in right and left lower lobes Pulmonary nodules in the left upper lobe (3 mm), right lower lobe (3 mm), right lower lobe (6 mm), and left lower lobe (4-5 mm).

Seven years after the initial presentation, surveillance neck ultrasound demonstrated a new nodule which biopsy confirmed to be tall cell papillary thyroid cancer recurrence. She was treated by total neck dissection (injuring her left recurrent laryngeal nerve and necessitating a temporary tracheostomy) and external beam radiation therapy for six weeks. At this time lung nodules were identified and thought to possibly be metastases (Figure 1).

Eight years after initial presentation, the patient underwent a prophylactic bilateral mastectomy because she had discharge from her breasts two years after stillbirth and many concerning areas on mammography. On surgical pathology, an invasive ductal papilloma was identified.

**Figure 2:**
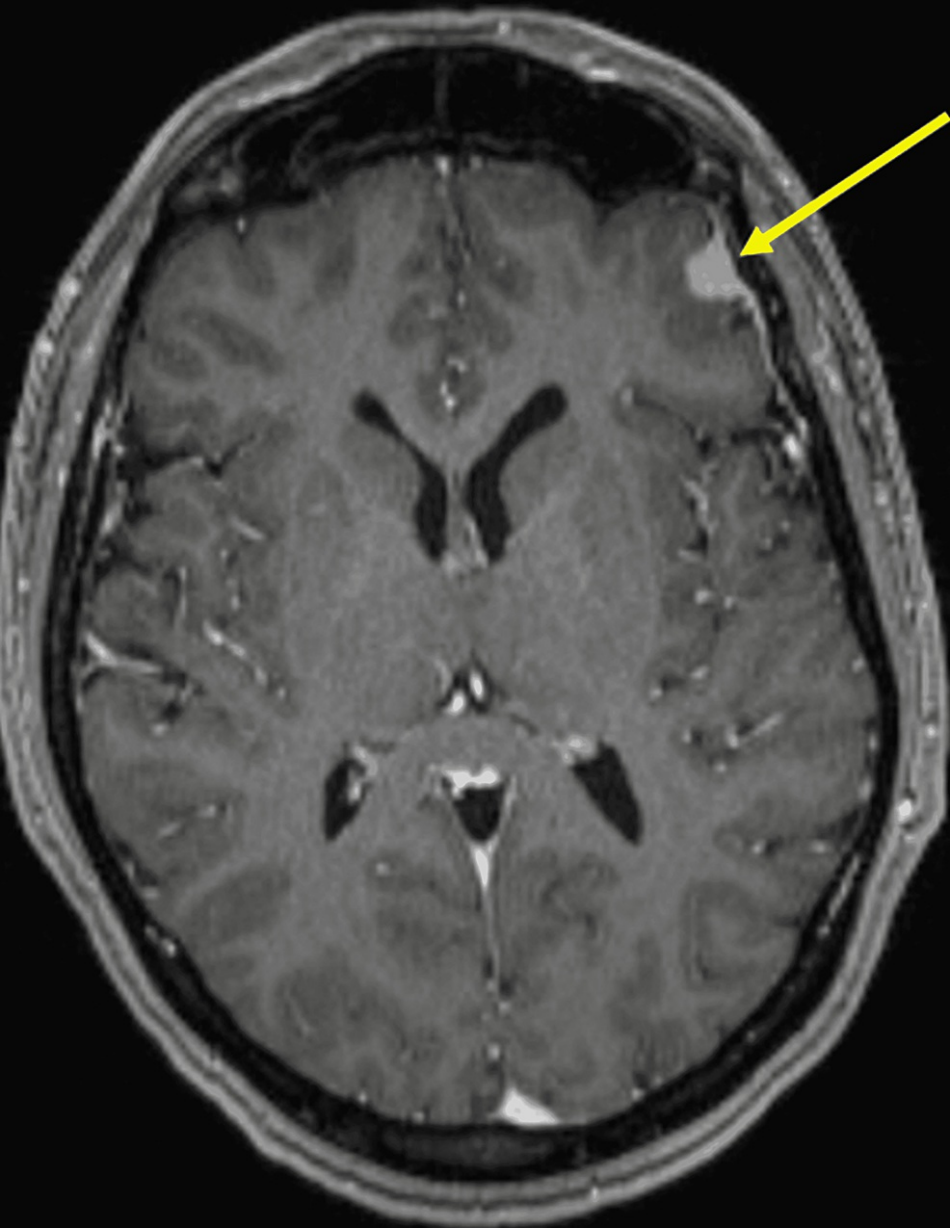
Brain MRI showing a left frontal extra-axial mass (1.0 x 0.8 cm) confirmed to be a meningioma on pathology

Ten years after the initial presentation, she successfully had a healthy child after leiomyoma removal by hysteroscopy. Twelve years after initial presentation, brain MRI found a T1 contrast-enhancing 1.9cm extra-axial mass, which was resected and confirmed to be a grade II meningioma on pathology (Figure 2).

**Figure 3:**
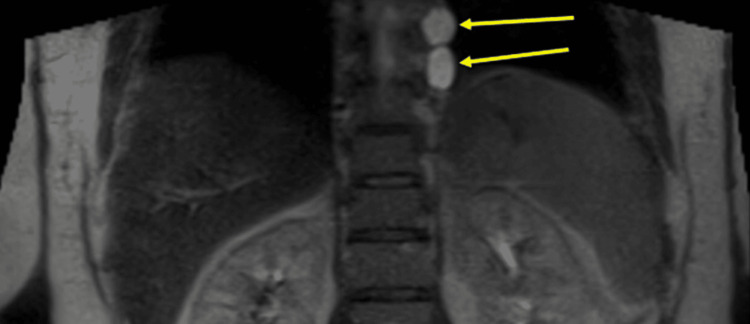
Full-body MRI showing nerve sheath tumors on the left from levels T4 to T8 T2 hyperintense paraspinal non-enhancing lesions on the left from levels T4-T8. The largest is at the T7 level and measures 2.0 x 1.4 cm, previously 2.2 x 1.9 cm.

**Figure 4:**
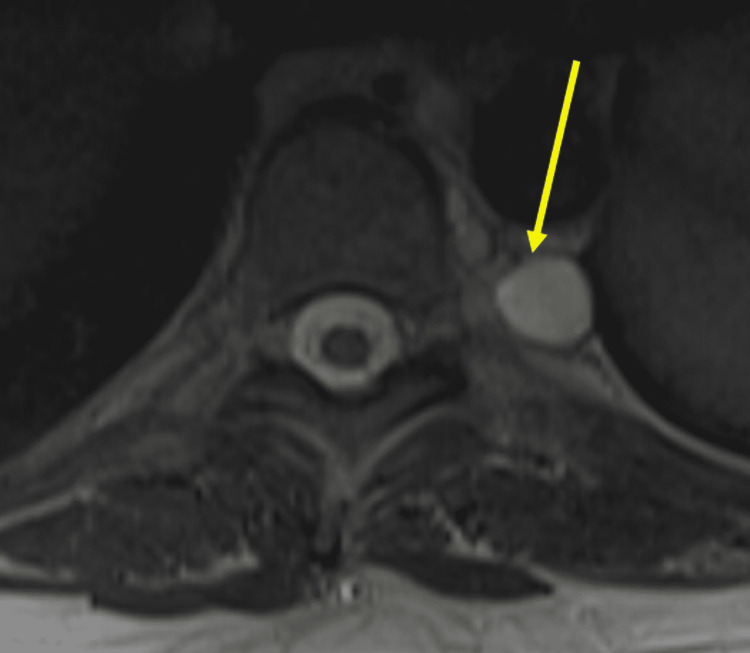
Full-body MRI showing the largest nerve sheath tumor at the level of T7 T2 hyperintense paraspinal non-enhancing lesions on the left from levels T4-T8. The largest is at the T7 level and measures 2.0 x 1.4 cm, previously 2.2 x 1.9 cm.

**Figure 5:**
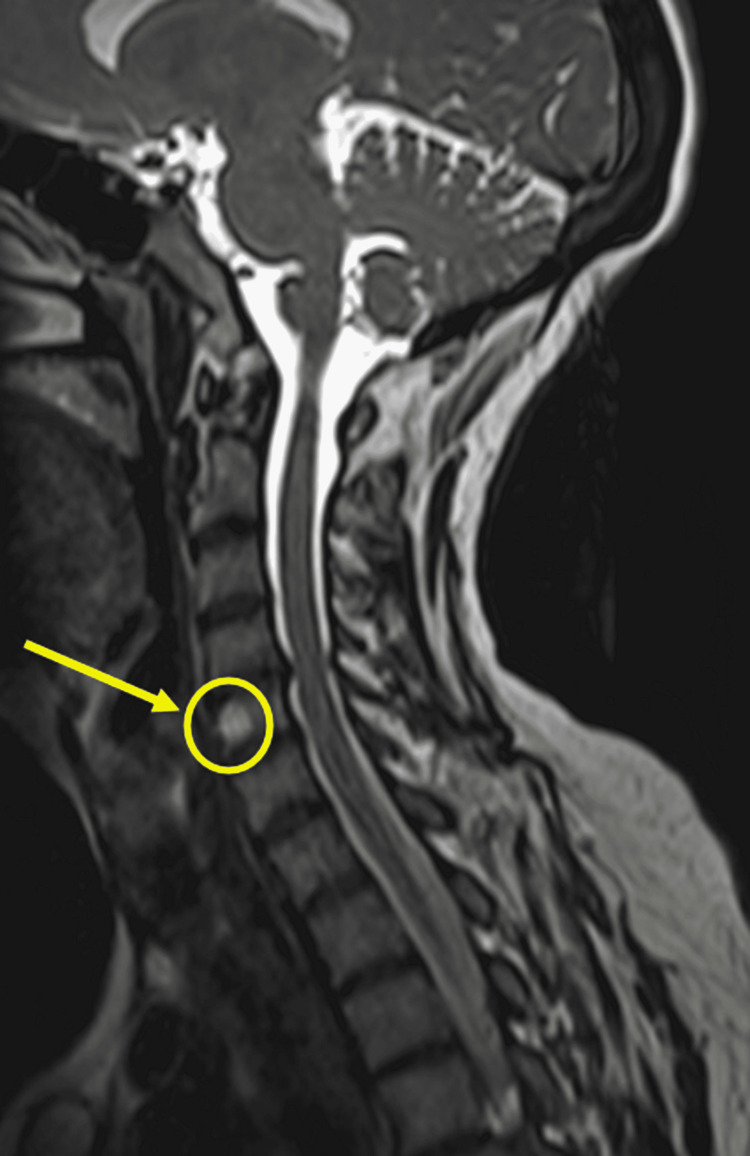
Full-body MRI showing vertebral hemangioma at level of C5 C5, L3, and L5 vertebral body interosseous hemangiomas. L3 and L5 hemangiomas are not shown in this image.

The patient is currently fourteen years out from the initial presentation. Due to her tumor history, she receives yearly colonoscopies and full-body MRIs. Her annual colonoscopies identify new polyps each time. The patient's yearly full-body MRIs have found her lung nodules to be stable (not growing). They have also found new tumors including nerve sheath tumors (Figures 3, 4) and hemangiomas (Figure 5) throughout her spine.

The patient has received RNA genetic testing of 91 cancer-related genes with +RNA insight from Ambry genetics which found no mutations except for a heterozygous *NTHL1* gene mutation (pq90 and pq287).

## Discussion

The tumor suppressor gene, *NTHL1*, plays an integral role in DNA base excision repair as it encodes DNA glycosylase [9]. The clinical manifestations of homozygous (biallelic) loss of function mutations in the *NTHL1* gene are better understood in the literature and are known to result in NTS. NTS is inherited in an autosomal recessive manner and is more commonly associated with an increased risk for breast cancer, colorectal cancer, and adenomatous polyposis [1]. Ovarian cancer, bladder cancer, meningioma, and other malignancies have also been presented due to biallelic *NTHL1* mutations [10]. Biallelic mutations of the *NTHL1* gene have been almost exclusively reported in the literature, but the risk for tumorigenesis in heterozygotes is unclear [1]. To our knowledge, there are only a handful of reports describing the formation of tumors in *NTHL1* heterozygotes [1,8,11], but the research is limited, and large population studies are needed to determine a more accurate prevalence. The prevalence of homozygous *NTHL1* mutations is one in 144,000 [1].

The patient presented in this case report underwent 91-gene genetic testing, which found no significant mutations except a heterozygous *NTHL1* gene mutation (pq90 and pq287). It is important to note that a genetic cause not identified in this 91 gene panel may be the cause of her tumors and that her *NTHL1* mutation may not be associated with her tumors. A population-based study in Finland of 1,333 breast cancer patients did not find *NTHL1* pq90 mutations to increase breast cancer risk [12]. Out of 488 patients with colorectal cancer, a Barcelona study found two to have heterozygous *NTHL1* pq90 mutations; one also had a meningioma, and the other had 26 colonic adenomas [13].

Fourteen years following the initial presentation, our patient developed both benign and malignant tumors throughout her body including a GIST, appendiceal carcinoma, pilocytic astrocytoma (similar tumor type to prior case report), several necks and lung nodules, uterine leiomyoma, recurrent thyroid cancer, invasive ductal papilloma (not associated with *NTHL1* mutations in Finland study), meningioma (found in Barcelona study), nerve sheath tumors (found in prior case report), hemangiomas (found in prior case report), and recurrent colon polyps (found in Barcelona study). Here, we present the development of tumors found in some locations not described in the associated literature.

It is well understood that having a biallelic mutation of the *NTHL1* gene increases the risk for tumorigenesis, but in heterozygotes, these findings are just starting to be unearthed. Previous studies on *NTHL1* gene mutations define heterozygotes as having no increased risk for cancer, and there are no current screening recommendations [1]. We present this case to add to the growing number of cases demonstrating tumor development in *NTHL1* heterozygotes, and for clinicians to consider appropriate screening (including possible full-body MRIs) if this mutation is found. Repeat full-body MRIs are the gold standard surveillance method for patients identified to have tumor syndromes that increase malignancy risk throughout the body [14]. Full body MRIs may be indicated in patients with *NTHL1* mutations and tumor history. Since colonoscopies provide better surveillance than MRIs for colonic malignancy, colonoscopies may also be indicated in these patients due to the association of *NTHL1* mutations with colorectal cancer. It must be said that this patient's tumors may not be associated with her being an *NTHL1* heterozygote.

## Conclusions

Homozygous mutations in the *NTHL1* gene are known to cause NTS, but more recently, heterozygous mutations have clinically manifested similarly, contrary to much of the associated literature. Our patient was found to have a single heterozygous mutation in the *NTHL1 *gene following comprehensive genetic testing and developed both benign and malignant tumors over 14 years. To our knowledge, this case presents the first evidence of tumorigenesis in locations different than what is known for NTS and another example of profound tumor growth in *NTHL1 *heterozygotes. Further investigation of heterozygous *NTHL1* variants is warranted to understand its role in tumorigenesis and call for screening consideration after diagnosis.
